# Development, Fabrication and Application of a Sectioned 3D-Printed Human Nasal Cavity Model for In Vitro Nasal Spray Deposition Studies

**DOI:** 10.3390/biomedicines14020329

**Published:** 2026-01-31

**Authors:** Anže Ličen, Jernej Grmaš, Špela Pelcar, Jurij Trontelj, Timi Gomboc, Matjaž Hriberšek, Gregor Harih

**Affiliations:** 1 Department of Biopharmaceutics and Pharmacokinetics, Faculty of Pharmacy, University of Ljubljana, 1000 Ljubljana, Slovenia; anze.licen@sandoz.net (A.L.);; 2 Sandoz Development Center Slovenia, 1526 Ljubljana, Slovenia; jernej.grmas@sandoz.net; 3 Laboratory for Manufacturing Science & Technology Biologics Lendava, Lek d.d., 9220 Lendava, Slovenia; spela.pelcar@sandoz.net; 4 Process Engineering and Computational Fluid Dynamics Laboratory, Faculty of Mechanical Engineering, University of Maribor, 2000 Maribor, Slovenia; timi.gomboc@um.si (T.G.);; 5 Laboratory for Integrated Product Development and CAD, Faculty of Mechanical Engineering, University of Maribor, 2000 Maribor, Slovenia

**Keywords:** nasal cavity model, 3D printing, fused filament fabrication, polypropylene, medical imaging, in vitro model, nasal drug delivery, biomedical engineering, nasal spray, nasal spray characterization, nasal cavity model deposition

## Abstract

In vitro models of the human nasal cavity are crucial for understanding the deposition dynamics of nasally administered drugs. Three-dimensional (3D) printing offers a powerful method for creating patient-specific, anatomically precise models for such experimental purposes. **Background/Objectives**: This study details the complete workflow for the development, design, and fabrication of a sectioned nasal cavity model intended for droplet deposition analysis of nasal sprays. **Methods:** A digital nasal cavity model was derived from medical imaging data and optimized for computer-aided design (CAD) operations. It was segmented into five therapeutically relevant regions: nasal vestibule, olfactory area, middle and upper turbinates, lower turbinate, and nasopharynx. Sections were 3D-printed in polypropylene for chemical compatibility, and a carbon fiber-reinforced fixation frame ensured precise alignment and airtight assembly. **Results:** Functional validation confirmed the model’s functional relevance through comparative deposition studies using automated actuation and high-performance liquid chromatography (HPLC) based regional quantification. Two devices with distinct spray characteristics (characterized separately by laser diffraction, plume geometry, and spray pattern imaging) were tested under varied administration conditions. The study demonstrated the model’s ability to discriminate between products, establishing a solid foundation for future investigations incorporating additional variables. **Conclusions:** Overall, the developed methodology provides a cost-effective and replicable platform for producing anatomically accurate, sectioned nasal cavity models. The newly developed in vitro system is well suited for detailed, region-specific analysis of nasal spray deposition, offering a valuable tool for pharmaceutical research and development.

## 1. Introduction

The intranasal route has emerged as a compelling alternative for drug delivery, enabling both systemic and targeted central nervous system (CNS) therapy through a non-invasive pathway that bypasses the blood–brain barrier and avoids first-pass hepatic metabolism [1,2].

As pharmaceutical applications for nasal delivery continue to expand, particularly in the development of vaccines and the administration of high-molecular-weight peptides and proteins [3,4,5], understanding and predicting regional drug deposition within the nasal cavity have become increasingly critical [6].

The nasal cavity is a complex structure with a volume of 15–20 mL, surface area of ~150 cm^2^, and length of 12–14 cm [7,8]. It begins at the vestibule, unsuitable for systemic absorption, followed by the respiratory region (~130 cm^2^) formed by turbinates—the main site for systemic uptake. Above lies the olfactory region (~15 cm^2^), critical for nose-to-brain delivery [8].

Important physiological and administration factors that impact deposition within nasal cavity are, but not limited to, mucociliary clearance, breathing, administration insertion position of nasal spray device (depth, angle), and head position [9,10,11,12,13]. Classical in vitro QC tests like droplet size distribution, spray pattern and plume geometry [14] are robust but do not address these factors, since they are performed in open air, lacking anatomical constraints and often showing poor in vivo correlation.

While in vivo assessment of nasal deposition using gamma scintigraphy, PET, or MRI can provide valuable insights [15,16], these methods pose ethical challenges by exposing volunteers or patients to drugs, raise safety concerns, and are often costly and insufficiently robust.

In contrast, testing with artificial nasal cavity models offers an affordable, safe, and highly reproducible alternative to both classical in vitro testing as well as in vivo studies. These models allow controlled, repeatable experiments in analytical laboratory environment without ethical constraints, making them an efficient tool for studying deposition patterns under conditions that closely resemble the in vivo environment.

Most recent FDA GDUFA Science and Research Report 2024 further emphasizes the importance of mechanistic nasal deposition modelling through internal projects and university granted contracts [17]. In these studies, they have combined anatomical nasal casts with Computational Fluid Dynamics (CFD) and pharmacokinetic approaches to predict regional deposition for nasal powders and sprays.

The fabrication of these complex anatomical structures has shifted largely towards high-resolution additive manufacturing technologies, specifically stereolithography (SLA) and PolyJet printing. Unlike standard Fused Deposition Modeling (FDM), which often yields porous structures with staircase artifacts, SLA utilizes UV-curable resins to achieve layer heights below 50 µm, producing the watertight integrity and smooth surface finishes essential for simulating mucosal surfaces [18,19]. This precision is paramount, as even minor surface roughness or manufacturing deviations in the narrow, tortuous meatuses of the nasal cavity can artificially alter airflow turbulence and subsequent drug deposition patterns [20].

However, material selection presents a critical dichotomy between geometrical fidelity and chemical stability. While SLA offers superior resolution, standard photopolymer resins are prone to leaching uncured monomers and photo-initiators into extraction solvents, which can result in interfering peaks during HPLC analysis and compromise the quantification of drug recovery [21]. In contrast, FDM printing, despite its lower resolution, offers the distinct advantage of processing chemically inert thermoplastics such as polypropylene (PP) [22]. The use of PP eliminates the risk of leaching and drug adsorption, ensuring that the model remains chemically neutral during rigorous pharmaceutical testing, a feature that is often challenging to achieve with commercially available UV-curable resins [23].

In parallel to technical advances also structural complexity of models increased over last few decades. Early designs were basic constructs, often glass or plastic tubes with two openings and a connected sinus cavity [24]. Later versions introduced simulated turbinates but lacked dismantlable sections and paranasal sinuses, limiting their relevance for realistic deposition studies [25,26,27]. More sophisticated models improved anatomical similarity by incorporating turbinate and olfactory regions, typically derived from CT scans and manufactured using stereolithography [20,28,29,30]. However, these remained incomplete, omitting critical structures such as paranasal sinuses and ethmoid regions.

The most advanced category now replicates full nasal and sinus anatomy using CT or MRI data combined with high-resolution 3D printing [31,32,33,34,35]. These models vary in dismantling approach: some divide the cavity into four axial sections for precise regional analysis, while others integrate separate maxillary and ethmoid sinus casts for sinus drug delivery studies.

Despite the high anatomical fidelity achieved by modern 3D-printed casts, a significant limitation persists in the source data utilized for model generation. The vast majority of existing literature relies on geometries derived from single-subject CT or MRI scans, often selected arbitrarily or based on specific pathologies [36]. While these patient-specific models are valuable for personalized medicine, they fail to account for the substantial inter-subject variability inherent in human nasal airway dimensions, which significantly influences deposition patterns [37]. Consequently, data generated from a unique anatomical instance may not be accurately extrapolatable to a broader patient group. There remains a distinct scarcity of studies utilizing 3D-printed models based on statistically averaged or “idealized” nasal geometries, which would provide a more robust, standardized platform for evaluating device performance representative of a median population rather than an isolated individual [38].

Designing a nasal cavity model requires balancing engineering precision, anatomical fidelity, and usability for high-throughput testing. Key steps include selecting representative geometry, segmenting into clinically relevant regions, and ensuring structural integrity for printing. Materials must be chemically inert for wide range organic polar and non-polar solvents compatibility and robust for repeated handling. The model should allow rapid assembly, cleaning, and fraction collection, supported by fixation frames for alignment and airtight sealing.

Functional validation is essential to ensure that the model is not only anatomically accurate, but also functionally relevant. It confirms that the system can discriminate between devices with different spray characteristics under controlled conditions. This is achieved through established analytical techniques such as laser diffraction, spray pattern imaging, and HPLC-based regional dose quantification. Rather than being an isolated technical exercise, validation defines the model’s role in shaping future development strategies. A validated platform enables consistent, reproducible data generation, forming the basis for comparative studies, device optimization, establishment of in vitro in vivo correlations and integration with computational approaches such as CFD. By establishing reliability and standardization, the model can become a practical development tool for advancing nasal drug delivery science and even a supporting tool as part of regulatory submissions to waive in vivo studies. This study applies a systematic, stepwise approach using advanced 3D printing to create a dismantlable, anatomically precise platform. The model is designed for reproducibility, ease of handling, and compatibility with quantitative analysis, serving as a foundation for testing nasal devices under varied physiological conditions and enabling integration with CFD-based digital twin development.

## 2. Materials and Methods

### 2.1. CAD Model Generation and Sectioning

The foundation of the physical model was a digital representation of an average human nasal cavity geometry developed by Brüning, Hildebrandt [39]. The anatomical geometries in this study were derived from 25 symptom-free adult subjects (age range 17–57 years; mean 37 years; 6 male; 19 female). Because ethnicity data were not recorded in the retrospective dataset, this information is unavailable. Nonetheless, the sample reflects a typical adult patient population from Central European. As a result, the anatomical findings and derived geometries are generalizable primarily to healthy adults without nasal pathology or alterations of the nasal anatomy, while anatomical variation associated with specific ethnic backgrounds, pediatric patients, or elderly individuals may not be fully represented. The average geometry was provided as an STL (Stereolithography) file. This file format represents the 3D surface geometry of an object using a mesh of triangular facets. Such models are typically generated from medical imaging techniques like Computed Tomography (CT) or Magnetic Resonance Imaging (MRI). The process involves acquiring a series of 2D cross-sectional images, which are then processed using specialized software to segment the region of interest (the nasal cavity) from surrounding tissues. This segmented volumetric data is then used to reconstruct a 3D surface mesh, which is exported as an STL file. The provided STL file was validated and confirmed to be a closed, manifold mesh with 89,314 vertices and 169,484 faces, containing no geometric errors that would impede further processing.

**Figure 1:**
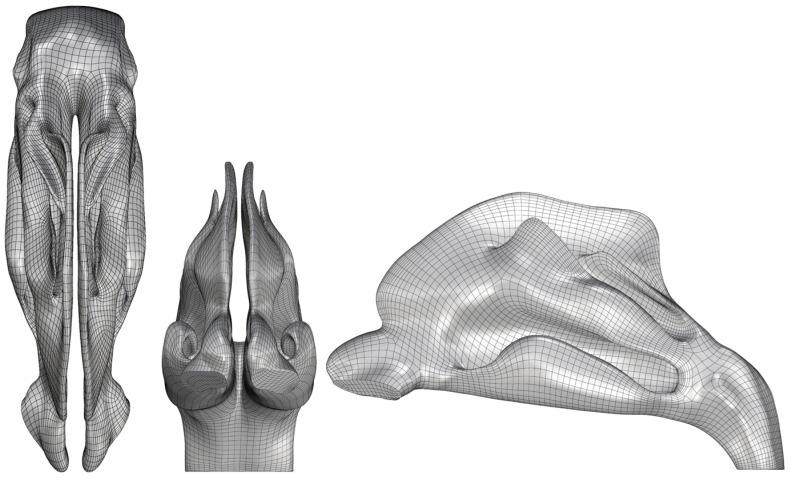
QuadMesh CAD nasal model in transverse (**left**), coronal (**middle**) and sagittal (**right**) plane.

While STL files are suitable for direct 3D printing, their triangulated mesh structure is difficult to modify using conventional CAD software. To enable advanced modeling operations such as sectioning and the addition of functional features, the STL mesh was converted into a quadrilateral mesh (QuadMesh) in Rhino 8 (Robert McNeel & Associates, Seattle, WA, USA), as presented in Figure 1. This conversion was performed using a QuadRemesh tool with a target quad count of 10,000 and an adaptive size of 50% to balance detail retention with topological regularity.

To ensure the geometric fidelity of the conversion process, a deviation analysis was performed, comparing the generated QuadMesh against the original STL model. The analysis quantified the geometric differences between the two surfaces, ensuring that the conversion did not introduce significant inaccuracies. This deviation was then compared to the typical accuracy of 3D reconstructions from CT scans, which ranges from 0.5 mm to 2 mm.

**Figure 2:**
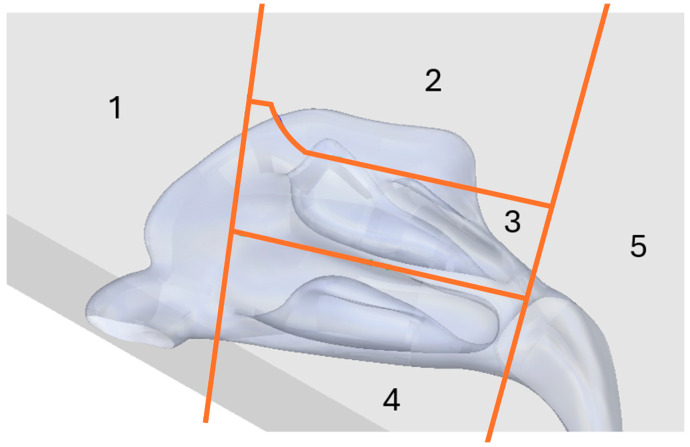
Schematic view of the nasal model sectioning in sagittal plane: Nasal vestibule (Region 1), Olfactory region (Region 2), Middle and upper turbinates (Region 3), Lower turbinate (Region 4), and Nasopharynx (Region 5).

The primary requirement for the experimental study was the ability to analyze spray deposition in distinct anatomical regions. To facilitate this, the QuadMesh model was sectioned into five sections as presented in Figure 2: Nasal vestibule, Olfactory region, Middle and upper turbinates, Lower turbinate and Nasopharynx.

The CAD preparation involved several steps. First, a solid cuboid was created around the nasal cavity model. A Boolean subtraction operation was then used to create a hollow block containing the internal geometry of the nasal cavity. This block was then sectioned using cutting planes defined by the anatomical boundaries. The entire sectioning strategy was designed to ensure that each part could be 3D printed without the need for support structures, thereby minimizing post-processing and material waste. Finally, the outer geometry of the blocks was optimized to reduce material usage during printing.

**Figure 3:**
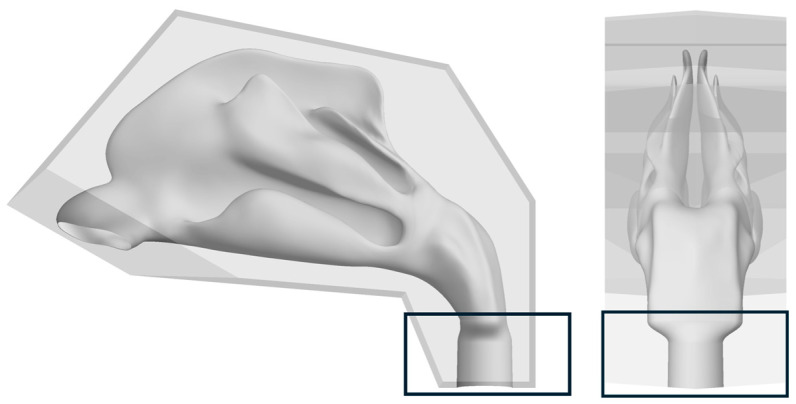
Nasopharynx (Section 5) with blended design for adapter in sagittal and coronal plane.

To integrate the model into the experimental setup, two key ancillary components were designed. A custom adapter was designed to connect the nasopharynx section of the model to an air pump hose. The design features a smooth, blended transition to minimize its influence on the airflow dynamics within the nasal model and ensure a secure, airtight seal (Figure 3).

**Figure 4:**
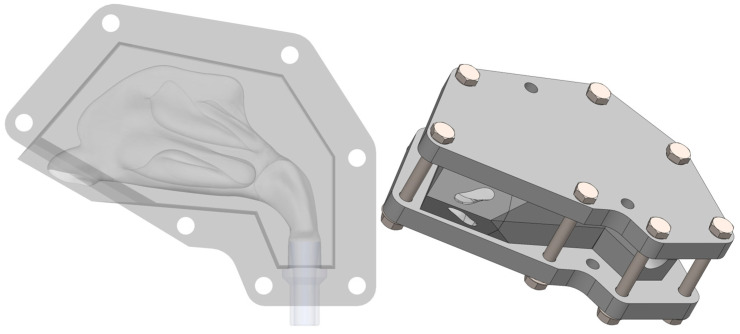
Fixation device design.

Fixation Device (Figure 4): A two-part frame was designed to hold the five nasal cavity sections in precise anatomical alignment and ensure a tight seal between them. The internal walls of the frame were designed with a 4° draft (taper), which matches a corresponding draft on the sectioned blocks. This feature uses a wedging effect to press the sections firmly together when clamped, enhancing both positioning accuracy and sealing. The frame includes strategically placed holes for bolting, ensuring uniform clamping force, and provides clear access to the nostrils for the experiments.

### 2.2. Material Selection and 3D Printing

The choice of materials was guided by the experimental requirements. Polypropylene (PP) was selected for the nasal cavity model sections. PP is a thermoplastic known for its excellent chemical inertness, making it highly resistant to the organic solvents (methanol and acetonitrile) used during extraction and HPLC analysis. It is also hydrophobic, cost-effective, and possesses a good balance of flexibility and toughness. PCTG CF, a composite of PolyCyclohexylenedimethylene Terephthalate Glycol reinforced with carbon fibers, was chosen for the fixation frame. Carbon fiber reinforcement provides significantly increased stiffness and dimensional stability compared to standard polymers, which is critical for maintaining the precise alignment of the model sections under clamping force.

All components were fabricated using a FlashForge Creator 4 FFF 3D printer (Zhejiang Flashforge 3D Technology Co., Ltd., Jinhua, China), which features an enclosed, heated chamber and a heated bed, essential for printing materials like PP. The sections were printed with a layer height of 0.1 mm to maximize surface detail and dimensional accuracy. A brim was used to improve bed adhesion and prevent warping. A gyroid infill pattern was selected for its nearly isotropic mechanical properties, ensuring uniform stiffness and helping the sections conform to each other for better sealing when clamped. The frames were printed with a honeycomb infill pattern to provide an optimal balance of structural rigidity and material efficiency.

### 2.3. Tested Nasal Spray Products

Two commercially available multidose delivery devices for nasal sprays (NS) were used in testing, NS A utilizing “VP3 design” (Aptar Pharma, Val-De-Reuil, France) and NS B utilizing “Classic standard” design (Aptar Pharma, Radolfzel, Germany), both manufactured by Aptar Pharma.

A total of twelve units were evaluated in this study, six units of each NS A and NS B. Within each delivery device type, one single unit with low observed variability between actuations at droplet size distribution (DSD) testing, was selected as representative for further characterization. Spray pattern, plume angle measurements, and in vitro nasal deposition studies were subsequently performed on these two selected units. Each unit was tested in triplicates under defined actuation velocity and acceleration conditions.

An aqueous solution formulation containing 150 mg/mL of the active pharmaceutical ingredient (API) was prepared for this study and filled in NSs. The API is a small, lipophilic model compound (log P ≈ 2), that is fully dissolved in the formulation. The compound exhibits a rapid onset of action and the ability to cross the blood–brain barrier. The API used in this study cannot be disclosed due to confidentiality constraints.

### 2.4. Analytical Characterization Methods

An automated actuation system Proveris Vereo^®^ NSx actuator operated through Viota^®^ software (Proveris Scientific, Hudson, MA, USA) was employed to ensure reproducible administration. Actuation parameters were derived from preliminary hand actuation studies for both NSs. Each actuation was initiated manually via the control interface.

#### 2.4.1. Droplet Size Distribution by Laser Diffraction

Malvern Panalytical Spraytec^®^ system (Malvern Panalytical Ltd., Malvern, UK), a laser diffraction droplet size analyzer, was used to measure the droplet size distribution (DSD). Measurements were performed at a 30 mm distance from the nozzle tip with an acquisition rate of 1 kHz. Data was recorded for 300 ms after light transmission decreased to 95% of the baseline value and for 50 ms before the transmission trigger.

The distribution was characterized by Dv10, Dv50, and Dv90, representing the droplet diameters at 10%, 50%, and 90% of the cumulative volume distribution, respectively. The distribution width was expressed as the span, calculated according to Equation (1):

Span = (Dv90 − Dv10)/Dv50(1)

#### 2.4.2. Spray Pattern and Plume Geometry by Non-Contact Laser Sheet Methods

A Proveris SprayVIEW^®^ system operated through Viota^®^ 8.1.1.17 software (Proveris Scientific, Hudson, MA, USA) was used for all experiments. All measurements were performed at a fixed distance of 30 mm between the device nozzle and the imaging system.

For spray pattern (SP) analysis, the quantitative parameters determined included the maximum diameter (Dmax, mm), minimum diameter (Dmin, mm), area (mm^2^) and Ovality (Dmax/Dmin). For plume geometry (PG) analysis, plume angle was determined.

#### 2.4.3. HPLC Method for Quantification of Deposited Dose Fractions

After each actuation, the nasal cavity model was dismantled, and the deposited drug from each region was extracted and quantified by high-performance liquid chromatography (HPLC). Analyses were performed on a Waters UHPLC system with a UV detector set at 215 nm. Separation was achieved on a C18 column (100 mm × 2.1 mm, 1.7 µm) maintained at 40 °C. The mobile phase consisted of (A) water with 0.1% phosphoric acid and (B) acetonitrile, using a gradient elution: 0–4 min, 5–30% B; 4–6.5 min, 30–55% B; column flushing and re-equilibration. The flow rate was 0.5 mL/min, and the injection volume was 5 µL. The method was checked for selectivity, accuracy, repeatability, and linearity.

### 2.5. Determination of Actuation Parameters

Actuation parameters were derived by comparing the DSD obtained from manual actuations with those produced by four automated actuation profiles. Eight adult volunteers (four women and four men) participated to ensure representative results. Each participant was provided with instructions how to actuate and performed three manual actuations per device unit. In parallel, three automated actuations per device unit were performed at four actuation velocities (25, 30, 40, and 60 mm/s) with a starting acceleration of 5000 mm/s^2^. Selection of the most appropriate actuation profile was based on achieving the most comparable Dv50 value between manual and automated actuation.

### 2.6. In Vitro Drug Deposition Study Methodology

**Figure 5:**
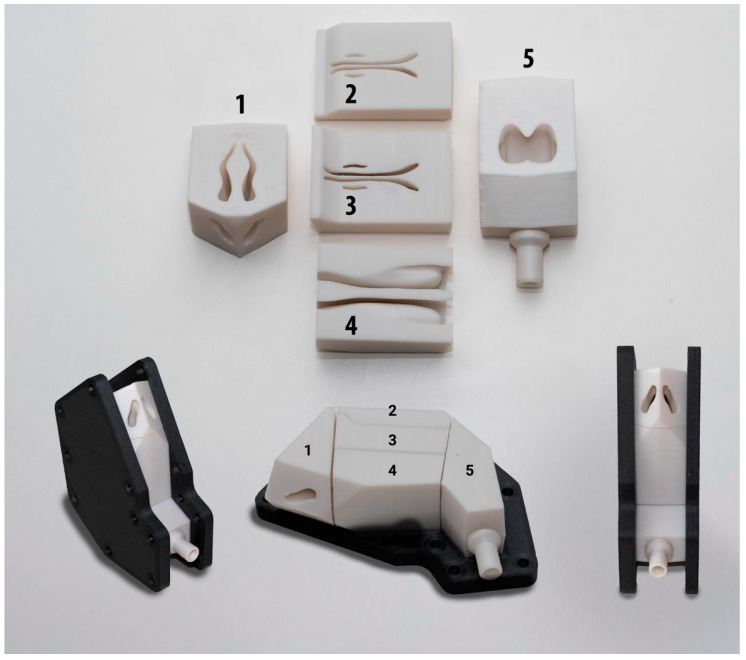
Assembled 3D-printed nasal cavity model in the fixation device and individual parts of the model. Nasal vestibule (Region 1), Olfactory region (Region 2), Middle and upper turbinates (Region 3), Lower turbinate (Region 4), Nasopharynx (Region 5).

Deposition experiments were conducted using the setup described above. A 3D-printed anatomical nasal cavity model was used to evaluate vitro deposition patterns. The model was segmented into five regions of interest, as described in Section 2.3, and is presented in Figure 5.

Each NS was actuated at three administration angles (60°, 45°, and 35°) while keeping head angle in position 0°. Although the newly developed nasal cavity model enables integration with controlled airflow generation for breathing simulation, all experiments were conducted under static airflow conditions. Prior to each deposition experiment, the NS was primed three times using the automated actuator. The NS was weighed before and after actuation using an analytical balance (Mettler Toledo, Greifensee, Switzerland).

Following actuation, the nasal cavity model was carefully dismantled into five regions of interest. The deposited drug was extracted from each region by placing the corresponding part into a separate 400 mL beaker containing 50 mL of solvent (0.1% (*v*/*v*) Orthophosphoric acid). Each section was rinsed to ensure complete recovery of the deposited NS. A 1.5 mL aliquot of each extract was then transferred into an LC vial for HPLC analysis. Regional deposition was calculated relative to the expected chromatographic response, which was calculated from the declared delivered dose of the NS device, the known formulation concentration, and the peak area obtained from an external HPLC standard solution. Drug recovery was determined using a dose-based analytical approach. Expected response was compared with the sum of chromatographic peak areas quantified from all five nasal cavity model sections to determine total recovery. Before each next actuation, the nasal cavity model was rinsed with water, air-dried, and reassembled.

### 2.7. Setup for In Vitro Drug Deposition Study

**Figure 6:**
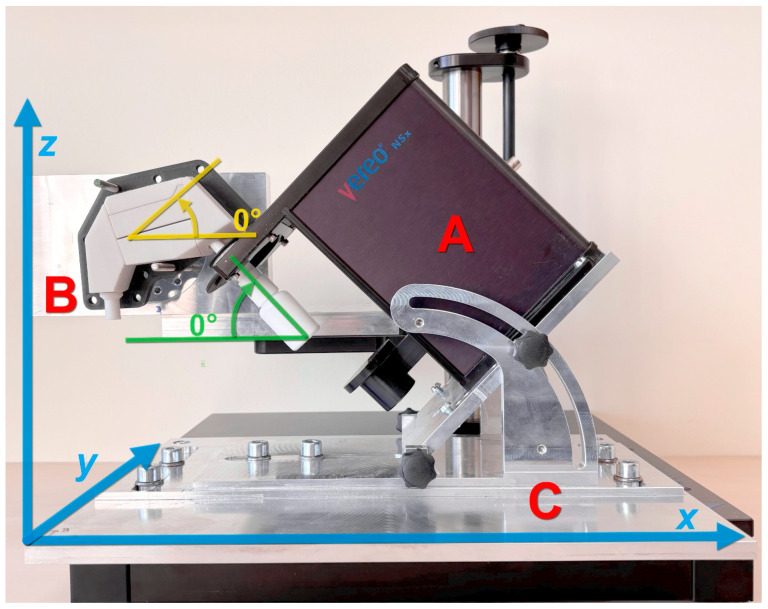
Experimental setup for deposition studies with: (**A**) holder with adjustable angle and actuator, (**B**) holder for nasal cavity model and (**C**) support plate.

The experimental setup is presented in Figure 6, and consisted of three main components, mounted on a commercially available stand from Proveris, modified to accommodate all three: (A) a Proveris Vereo^®^ NSx automatic actuator mounted in a holder with an adjustable administration angle (angle between the NS and the horizontal line, depicted in green), (B) holder for nasal cavity model with adjustable height (z axis, depicted in blue) and head angle position (angle between the model tilt and the horizontal line, depicted in yellow), and (C) a support plate enabling accurate actuator positioning along x and y axes (depicted in blue).

The setup enabled independent adjustment of the actuator angle and nasal cavity model orientation. Adjustment of the actuator angle simulated different NS orientation—simulating how patient would hold the NS. Actuator angle and administration angle are complementary. Tilting the nasal cavity model represented various head positions during administration. In all experiments presented, the nasal cavity model was maintained in a horizontal orientation corresponding to the natural upright head posture. 

### 2.8. Staistical Evaluation

To enable quantitative interpretation of deposition patterns, the nasal cavity was modeled as five anatomical regions arranged in a Cartesian coordinate system, as shown in Figure 2. The Middle and upper turbinates (Region 3) serve as the origin. The x-axis spans from the Nasal vestibule (Region 1, anterior) to the Nasopharynx (Region 5, posterior), while the y-axis connects the Olfactory region (Region 2, superior) and the Lower turbinate (Region 4, inferior). Each region’s deposited quantity contributes to a normalized vector originating at the origin, representing both direction and magnitude of deposition. For example, predominant deposition in the Nasal vestibule yields a vector pointing toward the positive x-axis, with length proportional to the deposited amount.

Two complementary multivariate analyses were performed. First, a two-factor MANOVA was applied to the x–y components of the deposition vector, enabling simultaneous evaluation of directional and magnitude effects. Second, because orientation is a circular variable, a MANOVA on transformed components [cosθ,sinθ] was conducted to isolate directional effects. In both cases, Device and Administration angle were treated as fixed factors, including their interaction. The significance threshold was set at 0.05.

In parallel, overall dose entry into the cavity was assessed via % Deposition recovered. This metric was analyzed using a two-way ANOVA with the same factor structure (Device, Administration angle, and interaction) at the 0.05 significance level. This combined approach allows interpretation of two critical aspects: (i) spatial distribution within the cavity and (ii) efficiency of dose delivery.

Statistical evaluation was conducted in Python 3.10.

## 3. Results

### 3.1. Digital Model Validation

The digital workflow successfully produced a high-fidelity QuadMesh model suitable for CAD operations. The deviation analysis comparing the QuadMesh to the original STL model revealed a maximum deviation of +0.34 mm and −0.32 mm. The vast majority of the model surface exhibited a deviation of less than 0.05 mm, as indicated by the color map analysis. This level of geometric accuracy is significantly smaller than the typical error range (0.5 mm to 2 mm) inherent in the initial 3D reconstruction from medical imaging data, confirming that the mesh conversion process preserved the anatomical fidelity of the original model.

### 3.2. 3D Printed Components Validation

#### 3.2.1. Nasal Cavity Sections

The five sections of the nasal cavity model, printed in PP, exhibited excellent detail and a smooth surface finish due to the 0.1 mm layer height. Complex anatomical features, such as the turbinates, were accurately replicated. The gyroid infill and wall configurations provided sufficient stiffness, and no significant warping or printing defects were observed, allowing for a seamless fit during assembly. To assess the reproducibility of the fabrication process, a total of five complete nasal cavity models were manufactured. All units were produced using high-grade Polypropylene filament under identical processing parameters. Specifically, low print speeds were maintained to ensure optimal layer cooling and surface definition. Post-fabrication, the dimensional accuracy of the printed sections was verified by measuring key external geometric features and connection interfaces using a digital caliper. All measured dimensions across the five replicates fell within a tolerance of ±0.1 mm, which corresponds to the printer’s layer resolution. While a full metrological analysis (e.g., via 3D scanning) was not performed to verify the complex internal mucosal topology, the high consistency of the external dimensions, achieved through the use of industrial-grade equipment and strict parameter control, provides strong evidence of geometric reproducibility and structural fidelity compared to the CAD data.

#### 3.2.2. Fixation Device

The fixation frames, printed in PCTG CF, were exceptionally rigid and structurally sound, showing no signs of deformation. The honeycomb infill pattern provided the necessary stiffness while keeping the parts relatively lightweight.

### 3.3. Final Assembled Apparatus

The assembly process was straightforward and effective. The 3D-printed nasal sections fit snugly into the slots of the fixation device. When the two halves of the frame were bolted together, the 4° drafted walls created a strong clamping force that secured all sections and created a tight seal between them. The final assembled apparatus was structurally robust, with the nostrils fully accessible and the nasopharynx adapter providing a secure connection for the air pump hose. The complete assembly mounted securely to the positioning device, creating a stable platform ready for the deposition experiments.

### 3.4. Actuation Parameters and Characterization of NS

Each unit was actuated manually 24 times and automatically 12 times, resulting in a total of 432 actuations (288 manual and 144 automated). Determined actuation parameters reflecting realistic user conditions were set to 40 mm/s for NS A, and 30 mm/s for NS B, with a starting acceleration of 5000 mm/s^2^ for both.

#### DSD, SP and PG

Six units of NS A and six units of NS B were evaluated. Based on overall performance, Unit 3 from Device A and Unit 2 from Device B were selected for subsequent experiments, as they demonstrated representative DSD for each device type and reproducible actuation. The mean Dv10, Dv50, Dv90, and span values for selected units are summarized in Table 1. For NS A, mean Dv50 value is 40 µm, while NS B showed smaller droplets, with Dv50 mean value 31 µm. The mean span values were 1.60 for NS A and 1.36 for NS B.

**Figure 7:**
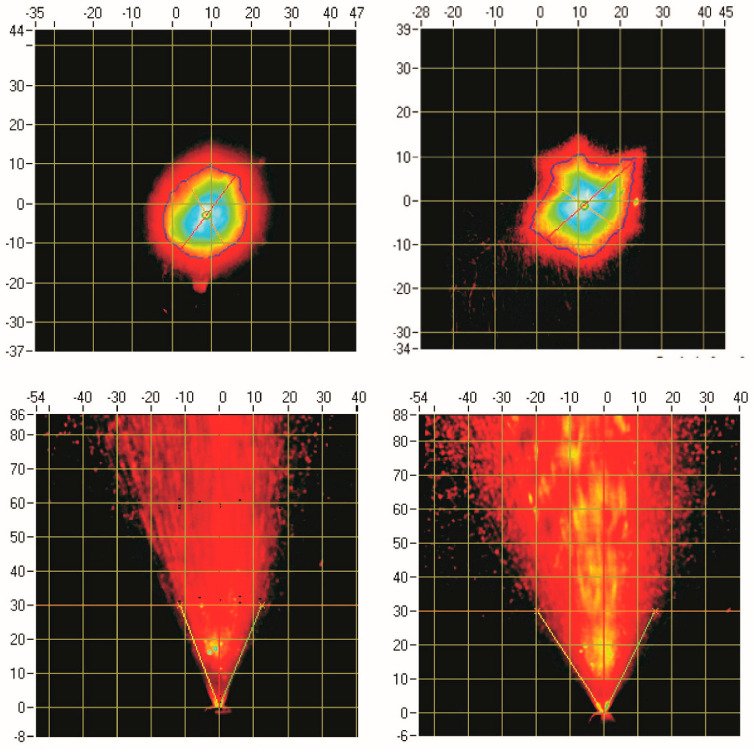
SP and PG for the NS A (**left**) and NS B (**right**). Scale grid is in mm.

SP analysis of NS A showed mean maximum and minimum diameters of 24 mm and 20 mm, respectively, and a spray area of 377 mm^2^. NS B exhibited a slightly broader spray, with Dmax and Dmin values of 28 mm and 20 mm, respectively, and area of 407 mm^2^. PG analysis showed plume angles of 44° (front view) and 48° (perpendicular 90° view) for NS A, and 61° and 58° for NS B, respectively. Figure 7 presents the SP and PG of NS A (left) and NS B (right), with color-coded intensity distribution mapped on a grid.

### 3.5. In Vitro Drug Deposition Study

**Figure 8:**
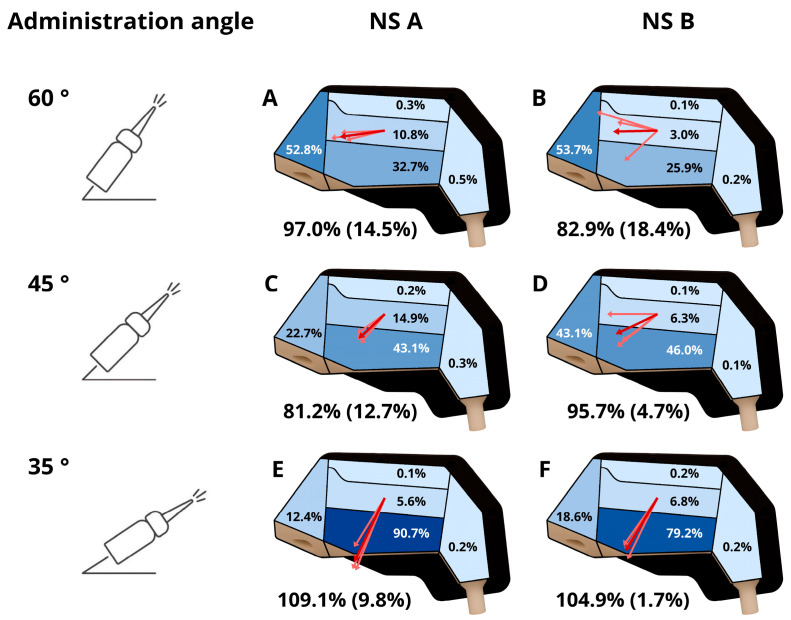
Results (**A**–**F**) for average deposition in each region of interest in nasal cavity model at different administration angles (60°, 45°, 35°) and total average recovery with RSD.

The nasal deposition profiles (A–F) of NS A and NS B at different administration angles (60°, 45°, and 35°) are shown in Figure 8. At angle 60°, both sprays favored anterior deposition (A, B), while angle 45° promoted deeper penetration and greater deposition in the middle and upper turbinates (C, D). For both NSs, deposition shifted progressively toward the lower nasal regions as the angle decreased (E, F). Deposition in olfactory and nasopharynx was low in all experiments. The recovery of administrated dose was greater than 80% in all deposition studies. Recovery relative standard deviation (RSD) is presented in backets.

Two-way MANOVA on vector components (Table 2) showed a highly significant effect of angle on combined magnitude and direction (Pillai = 1.202, *p* < 0.0001), while device type was not significant (*p* = 0.569). Interaction between device and angle was significant (*p* = 0.007), indicating that spatial distribution patterns vary with device-angle combinations. Direction-only MANOVA analysis (Table 3) yielded similar findings: angle strongly influenced orientation (*p* < 0.0001), and the interaction term was significant (*p* = 0.001), whereas device remained non-significant. For overall dose recovery (Table 4), two-way ANOVA revealed that angle significantly affected % deposition recovered (F = 5.703, *p* = 0.018), while device did not (*p* = 0.805). The interaction term showed only a weak trend (*p* = 0.098).

## 4. Discussion

### 4.1. 3D Nasal Cavity Model Creation and Sectioning

This study successfully demonstrated a complete workflow for the design and fabrication of a sectioned, anatomically accurate human nasal cavity model for in vitro spray deposition analysis. The final apparatus meets all the primary requirements for the intended experiments, including geometric fidelity, chemical inertness, and region-specific analysis capability.

While the choice of FFF printing with Polypropylene was essential for ensuring chemical compatibility and accurate HPLC quantification, we acknowledge that this fabrication method is often associated with higher surface roughness compared to SLA-based models. However, the models in this study were fabricated with a fine layer height of 0.1 mm. Visual inspection of the printed Polypropylene sections revealed a high-quality surface finish with almost no visible layer lines or “staircase” artifacts, a result attributed to the effective layer-to-layer fusion characteristic of this material. It is also important to note that this 0.1 mm vertical resolution is comparable to standard settings used in many SLA anatomical reconstructions, which themselves are not perfectly smooth and contain geometric approximations. Furthermore, unlike dry powder or nebulized aerosol studies where micro-scale surface roughness can significantly alter boundary layer airflow, nasal spray deposition is predominantly governed by inertial impaction driven by the device’s plume geometry and actuation velocity [29,40]. Therefore, while surface texture remains a variable, the priority in this work was to eliminate the risk of leachate interference to ensure robust chemical mass balance, with the achieved surface quality deemed sufficient for aerodynamic relevance.

A critical step in our digital workflow was the conversion of the initial STL mesh to a QuadMesh. This enabled the complex CAD operations required for sectioning and adding functional features, which would have been impractical with the original triangulated mesh. The deviation analysis confirmed that this conversion was achieved with minimal loss of anatomical accuracy, ensuring the geometric integrity of the model was maintained throughout the design process.

The design of the model itself incorporated several key features to enhance its experimental utility. As detailed in the next chapter, the first was sectioning the cavity into five distinct anatomical regions. Furthermore, the design was optimized for support-free 3D printing. This not only reduced material waste and post-processing time but also ensured that the critical internal surfaces of the nasal cavity were not marred by support structure artifacts, which could otherwise interfere with deposition patterns. The custom fixation device, with its drafted walls and stiff PCTG CF material, was instrumental in overcoming the challenge of sealing the multiple sections into a single, airtight unit, thereby ensuring the integrity of the simulated airflow.

### 4.2. Selection and Sectioning of Nasal Cavity Model

The use of a statistically averaged nasal cavity geometry provides a representative reference that avoids bias toward subject-specific extremes. As reported by Brüning and Hildebrandt [39], the averaged geometry exhibits airflow-relevant parameters that lie within the central range of values observed across individual anatomies, rather than reflecting unusually narrow or wide airways. In this study, the averaged model was therefore used to identify general deposition mechanisms and dominant influencing factors, rather than to quantitatively compare geometric metrics such as hydraulic diameter or airflow resistance with individual anatomies. Future studies may extend the present approach by applying the same experimental workflow to multiple representative geometries, enabling systematic evaluation of how deviations from average anatomical features influence nasal spray deposition.

Segmentation strategy was adapted and optimized from previously developed stereolithography-based models. Nasal models are typically divided into anatomically and physiologically relevant regions that reflect natural nasal geometry and therapeutic targets. Anterior (vestibule and valve), middle (turbinates), olfactory, and posterior regions are generally used for segmentation [20,30,40,41,42,43].

Our nasal cavity model was segmented into five regions (see Section 2.3 and Figure 5). This configuration provides improved deposition precision by isolating specific regions of interest, such as the olfactory region, which is critical for nose-to-brain delivery, or turbinates regions, playing important roles in local medical conditions such as congestion or allergy. Segmentation allows to assess potential of dose dripping (anterior region) or drug in small droplets which could enter posterior regions and deeper air pathways. Dismantlable systems such as ours enable regional deposition mapping, surface coating, cleaning, and enhanced reproducibility, whereas fixed models confine analysis to whole cavity deposition and limit interpretation of regional distribution [28,29,30,44,45,46,47,48].

### 4.3. NS Actuation Parameters and Characterization

The two nasal spray devices (NS A and NS B) were selected to exhibit distinct performance characteristics while remaining representative of established, off-the-shelf marketed designs. To ensure controlled and reproducible administration throughout all experiments, a dedicated study was conducted to determine appropriate actuation parameters. This approach ensured that automated actuation was physiologically representative, while minimizing the influence of manual variability and isolating the effects of device performance characteristics and administration angle.

Comparison of the characterization results demonstrated clear differences between NS A and NS B in terms of DSD and plume characteristics. NS A produced larger droplets overall, as reflected by higher Dv10, Dv50, and Dv90 values, together with a higher span (1.60), indicating a broader and less uniform DSD. In contrast, NS B generated smaller droplets with a narrower size distribution (Span 1.36). Measurable variability between individual units was observed for both device types; therefore, representative units with low intra-unit variability were selected to minimize their contribution to the total observed variability.

Spray pattern (SP) parameters also differed between the two devices. Although both NSs exhibited comparable minimum pattern widths (20 mm), NS B showed a larger maximum width and greater spray area (28 mm and 407 mm^2^) compared with NS A (24 mm and 377 mm^2^). In addition, NS B displayed higher ovality (1.45) than NS A (1.24), indicating a more elongated spray pattern for NS B and a more uniform, circular pattern for NS A. With respect to plume geometry (PG), NS B produced substantially wider plume angles (61°) than NS A (44°). The broader plume angle of NS B is consistent with its larger spray area and smaller droplet size distribution. These observations align with literature reports indicating that narrower plume angles are typically associated with larger droplets and more focused spray structures [10].

Overall, these results clearly demonstrate that NS A and NS B differ in both DSD and PG/SP behavior, confirming that the selected devices exhibit distinct performance characteristics and are suitable for evaluation of the nasal cavity model. NS A produced a more focused and regular plume accompanied by a broader droplet size distribution, whereas NS B generated a more dispersed, less symmetrical plume composed of smaller and more homogeneous droplets.

### 4.4. In Vitro Deposition Study

Conventional multidose nasal sprays typically deposit most of the delivered dose in the anterior and middle nasal regions, with limited ability to reach superior regions [49,50,51]. Previous studies have shown that NS A-type sprays deliver the majority of the dose to the anterior nasal cavity and result in minimal deposition in upper regions [14,29,52]. More recently, Fang et al. demonstrated that variations in plume angle and droplet size distribution (DSD) lead to clear, device-dependent differences in regional deposition within a 3D-printed nasal cavity model [19].

The deposition results obtained with the present nasal cavity model are consistent with these reports. Deposition in the olfactory and nasopharyngeal regions remained below 1% for both devices, confirming minimal penetration into the superior and posterior nasal regions. Instead, the majority of the dose accumulated in the vestibule and lower turbinate regions. Notably, NS B consistently produced higher deposition in the vestibule region than NS A, regardless of administration angle, reflecting the influence of its broader plume and smaller droplets on deposition behavior.

The influence of DSD, plume geometry (PG), and spray pattern (SP) on intranasal deposition has been widely discussed in the literature. While some studies report limited effects of droplet size alone, others show that larger droplets tend to deposit predominantly in the anterior nasal cavity, with reduced penetration into posterior regions [40,53,54]. Narrower plume angles facilitate passage through the nasal valve and support deeper penetration, whereas wider plumes promote anterior deposition and limit penetration [28,29]. Because narrower plumes are often associated with larger droplets, while wider plumes resemble a mist of smaller droplets, several authors have concluded that PG and SP are more informative predictors of nasal deposition than DSD alone [10].

In the present study, NS A produced larger droplets and a narrower, more concentrated plume (44°) compared with NS B (61°). These differences were directly reflected in the deposition results. Across all tested administration angles, NS B consistently showed higher deposition in the vestibule region, whereas NS A achieved greater deposition in the middle and upper turbinate regions. This behavior indicates deeper penetration for NS A and supports literature findings that plume geometry and spray pattern exert a stronger influence on deposition than droplet size alone.

In addition to plume characteristics, nasal model studies consistently identify administration angle as one of the most influential determinants of nasal spray deposition. Lower administration angles (approximately 0–30°) favor deposition in inferior regions, while angles around 30–45° improve penetration beyond the nasal valve and promote deposition in the middle and turbinate regions. In contrast, steeper angles (≥60°) predominantly result in anterior or vestibular deposition [20,29,30,55,56,57]. In these studies, administration angle is defined as the angle between the nasal spray device and the horizontal plane.

More recent work has further refined this understanding. Rigaut et al. demonstrated that olfactory deposition of dry powders can be increased by directing the device toward the olfactory region rather than toward the center of the nasal valve [58]. Similarly, Seifelnasr et al. showed that small adjustments in head position and nozzle orientation can substantially enhance central and olfactory deposition, identifying an optimal combination of a 45–60° backward head tilt from the supine position and a 5–10° nozzle angle (relative to the line normal to the nostril) to achieve olfactory deposition levels of approximately 23% [12,59].

Our regional analysis showed that the vestibule region received the highest deposition at an administration angle 60° for both devices. In contrast, lower turbinate region exhibited markedly higher deposition at 35°. At administration angle 45°, NS A produced greater deposition in middle and upper turbinates region than NS B, demonstrating better penetration for NS A. As deposition in lower turbinate region increased, deposition in vestibule and middle and upper regions decreased, demonstrating a shift toward deeper penetration with decreasing angle.

To complement regional analysis, we introduced an innovative quantitative approach that evaluates deposition as a whole rather than by individual compartments. Each deposition pattern was reduced to a normalized vector originating from the middle and upper turbinates, describing both magnitude and direction within the nasal cavity (Figure 8). At an administration angle of 60°, the vectors for both devices point predominantly toward the anterior direction, indicating dominant vestibular deposition. At 45°, the vectors shift posteriorly and slightly inferiorly, reflecting increased penetration into the middle and upper turbinate regions, particularly for NS A. At 35°, the vectors are strongly directed inferiorly, consistent with the pronounced increase in lower turbinate deposition and reduced anterior contribution.

This representation enables statistical evaluation of overall distribution using two-way MANOVA (Table 2 and Table 3). Results confirmed that administration angle strongly governs both magnitude and orientation (*p* < 0.0001), while device type remains non-significant. Interaction between device and angle was significant for pattern orientation (*p* = 0.001), indicating subtle differences in directional behavior. In parallel, % deposition recovered was analyzed by two-way ANOVA (Table 4), showing that angle significantly affects delivery efficiency (*p* = 0.018), whereas device differences are negligible (*p* = 0.805). Together, these complementary evaluations demonstrate that within tested space (two device types and administration angle), administration angle statistically significantly governs both intranasal distribution and delivery efficiency. Given that the two tested devices exhibited only minor differences in overall deposition behavior, future work should explore alternative device designs with fundamentally different spray characteristics or delivery mechanisms to achieve meaningful improvements in regional targeting and systemic delivery.

Deposition in the olfactory region remained consistently low for both devices (<1%), the highest values were achieved with NS A at angle 60°.The consistently low deposition observed in the olfactory region reflects a combination of factors rather than a limitation of the nasal cavity model geometry itself. This finding is consistent with literature reports indicating that conventional multidose nasal spray pumps are generally inefficient at targeting the superior nasal cavity due to their plume geometry and droplet kinetics [28,29,36,42].

Anatomical factors also contributed to this outcome. A retrospective visual analysis of the digital nasal cavity model was performed to assess the “line of sight” from the nostril to the olfactory region. This analysis showed that, for the statistically averaged geometry used in this study, a direct trajectory toward the olfactory slit is geometrically possible but highly restricted. Successful alignment requires not only an appropriate administration angle but also a specific lateral rotation of the device. In the experimental setup applied here, the roll angle was fixed at 0°. Consequently, even at administration angles that favor deeper penetration (e.g., 45°), the spray alignment may have been insufficient to fully bypass the nasal valve and turbinate restriction.

While individual patient anatomies may provide more accessible pathways due to biological variability, these findings suggest that for a standardized, averaged geometry, effective olfactory targeting requires either specialized delivery devices or more complex, multi-axial administration strategies that optimize both pitch and roll. Future studies should therefore consider incorporating controlled device rotation and alternative head positions (e.g., head down configurations) to further investigate these Fgeometric dependencies.

Another factor that may contribute to low olfactory deposition is the absence of simulated breathing during nasal spray actuation. Airflow has been shown to influence aerosol transport toward posterior and olfactory regions, and therefore the deposition results reported here likely represent conservative estimates for olfactory and posterior delivery. Nevertheless, multiple experimental and computational studies have demonstrated that, for conventional nasal spray devices, deposition is predominantly governed by plume geometry, droplet size distribution, and administration angle, whereas breathing conditions exert a comparatively minor influence during actuation. Future investigations incorporating physiologically relevant airflow profiles would allow more detailed evaluation of airflow-related transport effects [29,40,45,60,61,62].

Recovery values, calculated relative to the expected chromatographic response per actuation, were close to 100%, with some values marginally exceeding 100% (Figure 8). Recoveries above 100% reflect the combined effects of analytical variability and inherent variability in NS device delivery. Because recovery was normalized to the nominal delivered dose used to calculate the expected response, individual devices or single actuations delivering higher doses than declared may result in apparent recoveries above 100%, which is consistent with regulatory allowances for delivered dose variability (individual spray weights up to ±15% from the target value and mean delivered dose deviations up to ±10%) [63].

Higher mean recovery values were observed at an administration angle of 35°, and no systematic trend in total recovery was identified between devices. Variability, expressed as relative standard deviation (RSD), was lowest at 35°, particularly for NS B (1.8%). At steeper administration angles (60°), the spray impacts the anterior nasal region more rapidly, promoting liquid accumulation and nasal dripping. This effect likely contributes to slightly lower mean recovery values and increased variability, reflected by higher RSD values for both NSs. Importantly, total recovery inherently incorporates any losses occurring during the experiment, including residues on holders, connecting elements, or external surfaces (e.g., nasal dripping). The consistently high recovery observed across administration angles and devices therefore indicates that such losses were limited and confirms the robustness of the experimental setup. Further improvement of recovery could be achieved by incorporating a sealing element between individual model sections, which would reduce potential liquid losses at the junctions of the assembled parts.

In summary, we developed a novel nasal cavity model derived from a statistical shape model based on 25 subjects, as reported by Brüning and Hildebrandt [39], providing a statistically averaged nasal geometry representative of a Central European population. The model was sectioned into five clinically relevant anatomical regions and integrated into an experimental setup that enables numerically defined and highly reproducible nasal spray administration. The presented platform allows detection of deposition differences across administration angles and between devices (thru interactions), demonstrating its discriminatory capability. Although the nasal cavity model was not directly validated against in vivo measurements, its demonstrated discriminatory capability offers a meaningful form of functional validation. In addition, it supports the development and evaluation of next generation NS devices designed to more effectively target specific nasal regions. By minimizing patient-induced variability in delivery, the model facilitates robust performance testing during nasal spray development and offers clear relevance for regulatory assessment. Overall, this work introduces a comprehensive, versatile, and standardized experimental framework suitable for both academic research and industrial applications.

Future work should address current limitations related to static surface properties and the absence of dynamic physiological processes such as mucociliary clearance and nasal tissue compliance. Additionally, the inherent hydrophobicity of the PP surface highlights the need for developing an artificial mucus layer to better replicate in vivo conditions. Addition of the mucus layer could address the wettability of the surface, fixation, and retention of applied liquids and increase physiological relevance. Although the present study focused on quantitative recovery and regional deposition, visualization-based approaches using tracer or marker compounds could provide complementary qualitative insight into deposition patterns and potential post-deposition redistribution. Physiologically relevant airflow profiles would elucidate airflow-related influence on nasal deposition and could facilitate grater olfactory deposition. Use of the specially designed devices for olfactory delivery and lateral rotation (roll) of the device could improve deposition in superior regions. While substantial interindividual variability in nasal anatomy is well documented and may influence airflow and spray deposition, the use of a single statistically averaged nasal cavity geometry in this study provides a meaningful reference for identifying general deposition mechanisms and dominant influencing factors in Central European population. Future work may extend this approach by generating multiple representative nasal cavity models based on averaged geometries of distinct anatomical phenotypes or population subgroups, should such phenotypic patterns prove functionally relevant for nasal spray deposition. The experimental workflow established in this study is well suited for such an extension and could enable more robust, population-representative assessment of nasal drug delivery performance. Expanding the model library to include pediatric, geriatric, and pathological anatomies, exploring post-processing or multi-material printing to better emulate mucosal surfaces, and integrating the platform with CFD-based digital twin approaches represent meaningful research directions. The latter would have to incorporate a computational model of multiphase flow including administration device specific spray model with Lagrangian droplet tracking in turbulent flow conditions, which would, once validated by the results of the present study, enable a deeper insight into device and nasal cavity specific droplet dynamics and regional droplet deposition. These advances could further strengthen the predictive value of in vitro deposition assessments, increase translational relevance, and support the potential use of validated nasal models in regulatory strategies aimed at reducing reliance on in vivo studies

## 5. Conclusions

This work established a comprehensive and replicable methodology for the development, fabrication, and application of a sectioned 3D-printed human nasal cavity model. The aim was to create an anatomically realistic and practically deployable platform capable of supporting quantitative, region-specific evaluations of nasal drug delivery performance. This was achieved through a digitally driven workflow, beginning with the use of statistically averaged anatomical geometry and culminating in the fabrication of chemically inert polypropylene model sections supported by a carbon-fiber reinforced fixation frame, enabling precise alignment and airtight assembly.

The deposition results generated using two NSs with distinct plume and droplets characteristics confirm that the model can discriminate between device designs, administration angles, and their interaction, demonstrating its capability to detect clinically meaningful performance differences. For future testing, the implementation of NSs specially designed for focused delivery to upper nasal cavity regions should be considered for greater olfactory deposition. Furthermore, the statistically averaged geometry represents a key advancement over the predominantly single-subject models reported in prior literature. By improving population representativeness, the model supports standardized performance evaluation and enhances the potential for establishing clinically relevant in vitro–in vivo correlations and regulatory confidence. Clinically, this platform offers value for the development and optimization of nasal drug products targeting systemic absorption, local therapeutic action, or CNS delivery. The ability to simulate patient-induced variability in nozzle orientation and administration angle further aligns its use with real-world clinical and user behavior, supporting risk-based robustness assessments during pharmaceutical development or bioequivalence testing.

## Figures and Tables

**Table 1:** Result for DSD, SP and PG for NS A and NS B, presented as mean ± standard deviation. 0 means that SD is lower than 0.5.

NS	Dv10 [µm]	Dv50 [µm]	Dv90 [µm]	Span	Dmax [mm]	Dmin [mm]	Area [mm^2^]	Ovality	Plume Angle [°]/Perpendicular Positions
A	19 ± 0	40 ± 0	83 ± 0	1.60 ± 0.01	24 ± 0	20 ± 0	377 ± 5	1.24 ± 0.02	44 ± 2/48 ± 2
B	16 ± 0	31 ± 0	58 ± 0	1.36 ± 0.04	28 ± 0	20 ± 0	407 ± 2	1.45 ± 0.03	61 ± 1/58 ± 1

**Table 2:** Two-way MANOVA for direction and magnitude effect (vector x-y). * indicates an interaction between the two factors.

Effect	Pillai	F	df1	df2	*p*
Device	0.087	0.589	2	13	0.569
Angle	1.202	22.588	4	30	1.09 × 10^−8^
Device * Angle	0.449	4.339	4	30	0.007
Residual	1345.484	12			

**Table 3:** Two-way MANOVA for direction-only effect [cos θ,sin θ]. * indicates an interaction between the two factors.

Effect	Pillai	F	df1	df2	*p*
Device	0.212	1.543	2	13	0.250
Angle	1.011	15.339	4	30	6.17 × 10^−7^
Device * Angle	0.560	5.827	4	30	0.001

**Table 4:** Two-way ANOVA for % Deposition recovered. * indicates an interaction between the two factors.

Effect	Sum_sq	df	F	PR (>F)
Device	7.128	1	0.064	0.805
Angle	1278.817	2	5.703	0.018
Device * Angle	636.140	2	2.837	0.098
Residual	1345.484	12		

## Data Availability

The datasets presented in this article are not readily available because the raw data is the property of company Sandoz. Requests to access the datasets should be directed to Sandoz (jernej.grmas@sandoz.com).
